# Validation of the Children’s Separation Anxiety Scale – Parent Version (CSAS-P)

**DOI:** 10.3389/fpsyg.2022.783943

**Published:** 2022-04-05

**Authors:** Xavier Méndez, José P. Espada, Juan M. Ortigosa, José M. García-Fernández

**Affiliations:** ^1^Department of Personality, Assessment, and Psychological Treatment, Universidad of Murcia, Murcia, Spain; ^2^Department of Health Psychology, Miguel Hernández University, Elche, Spain; ^3^Department of Developmental Psychology and Teaching, University of Alicante, Alicante, Spain

**Keywords:** children, separation anxiety, psychometric adaptation, parents, assessment

## Abstract

The main objective of this research was to validate the parents’ version of the Children’s Separation Anxiety Scale (CSAS-P), which assesses separation anxiety symptoms in pre-adolescence, the stage with the highest incidence of anxiety disorder due to separation. In Study 1, 1,089 parents, those children aged between 8 and 11 (*M* = 9.59, *SD* = 1.11), 51.7% girls, were selected by random cluster sampling, who completed the CSAS-P to obtain the factorial structure. Exploratory factor analysis identified four related factors: Worry, Opposition, Calm, and Distress, which explained 42.93% of the variance. In Study 2, 3,801 parents, those children aged between 8 and 11 (*M* = 9.50, *SD* = 1.10), 50.2% girls, completed the CSAS-P, and their children completed the Children’s Separation Anxiety Scale (CSAS). The four related-factor model from Study 1 was validated by confirmatory factor analysis. The CSAS-P had adequate internal consistency (α = 0.84), temporal stability (*r* = 0.72), and invariance across children’s age and gender and the parent who completed the scale. Age and gender differences were small: older children scored higher on Worry and younger children on Distress; the girls scored higher on all factors. Small differences were also found depending on the parent who completed the scale without finding a clear pattern. Parents scored significantly lower than the child on all four factors of the scale. The results support the reliability and validity of the CSAS-P, an instrument that complements the child’s self-report in the framework of the multi-source assessment.

## Introduction

Separation anxiety disorder (SAD) in childhood is the child’s disproportionate and maladaptive anxiety when they are separated from their main caregivers, usually the parents, or when they anticipate separation. Anxiety manifests in the form of excessive concern for the well-being and health of the attachment figure or the child themselves (e.g., that the parents might suffer an accident); associated discomfort (e.g., stomachache and nausea at school); and opposition to separation (e.g., protests to prevent parents from going out at night by leaving them with a babysitter) (American Psychiatric Association, 2013).

It is estimated that up to 12% of children are diagnosed with an anxiety disorder (Essau et al., 2018), with SAD being the most prevalent one under 12 years of age (American Psychiatric Association, 2013). Along with several types of specific phobia, specifically, animal, blood-injection-injury, and environmental, SAD presents at an earlier age of onset, and most cases of SAD begin before the age of 12 (Beesdo et al., 2009), with a mean age of onset of 8 years (Keller et al., 1992). In an epidemiological study in which 29,699 children and adolescents were randomly selected, the prevalence of SAD was 5.3% and was more frequent in the 6-9 years (7.2%) and 10-14 years (5.5%) age groups, than in the 15-18 years group (3%) (Mohammadi et al., 2020).

The avoidance of situations that involve separation from attachment figures or withdrawal from home restricts the child’s social relationships, has a negative impact on family functioning, and causes problems with school attendance. Symptoms of separation anxiety and school fears are strongly linked (Orgilés et al., 2009). A significant proportion of cases of school refusal present SAD: 22.2% in the clinical population (Allen et al., 2010) and 10.8% in the community population (Egger et al., 2003). Students with symptoms of separation anxiety have worse social functioning (Gonzálvez et al., 2019) and higher rates of school absenteeism (Fornander and Kearney, 2020).

The comorbidity of SAD with other disorders is high, with rates of up to 86% (Shear et al., 2006), especially with generalized anxiety disorder (74%) and with specific phobia (58%) (Verduin and Kendall, 2003). SAD is not only associated with other anxiety disorders, but also with various disorders such as Gilles de la Tourette syndrome (Eapen et al., 2018). The presence of SAD in childhood predicts the same disorder in adolescence (13-19 years) (Bittner et al., 2007) and is a powerful risk factor (78.6%) for the development of psychopathology in early adulthood (19-30 years) (Lewinsohn et al., 2008). SAD increases the risk of many disorders, including panic disorder, depression, and substance abuse (Aschenbrand et al., 2003; Hayward et al., 2004; Biederman et al., 2007; Brückl et al., 2007). Concerning SAD in adulthood, 36.1% presented it in childhood, especially women (Silove et al., 2010).

The prevalence of SAD in childhood, its serious negative repercussions in the family, school, and social spheres, its high comorbidity, and the risk of psychopathology in adolescence and adulthood advise early detection and early treatment of the disorder. In the evaluation of anxiety disorders, questionnaires and scales are widely used for their ease of administration, correction, and interpretation. From the point of view of multi-source evaluation, it is recommended to complement the child’s self-report with the parents’ report, especially considering that parents are the most important source of information for the clinician in the evaluation of the child’s emotional problems (Achenbach et al., 1987; Kazdin, 1988). Concerning SAD, children report discomfort more precisely, while their parents report disruptive behaviors (Allen et al., 2010). Parents often complain that their child cries, has tantrums, follows them around the house like their shadow, sleeps with them, refuses to participate in extracurricular activities, and performs other behaviors that affect family functioning.

There are parents’ versions of generic scales that assess anxiety disorders in childhood, including the SAD: the Multidimensional Anxiety Scale for Children (MASC; March et al., 1997), the Screen for Child Anxiety-Related Emotional Disorder (SCARED; Birmaher et al., 1997), and the Spence Children’s Anxiety Scale (SCAS; Spence, 1997). However, these scales, widely used in epidemiological studies, include only a reduced set of SAD items. In clinical contexts, it is useful to have specific instruments that collect the relevant aspects of SAD and that help plan therapy based on the particular characteristics of the case. There are two instruments for parents: the Separation Anxiety Avoidance Inventory - Parent Version (SAAI-P; In-Albon et al., 2013) and the Separation Anxiety Assessment Scale - Parent Version (SAAS-P; Eisen and Schaefer, 2005). The 12 items of the SAAI-P are limited to evaluating avoidance behavior, omitting fundamental dimensions such as worry and discomfort. The SAAS-P allows a more comprehensive evaluation, but its 34 items mix symptoms with triggering events of the disorder and safety signals that reduce separation anxiety. Moreover, both instruments evaluate separation anxiety indistinctly in childhood and adolescence: 4-15 years (SAAI-P) and 6-17 years (SAAS-P), although its manifestations vary with age. In a classic study, Francis et al. (1987) found that separation nightmares were more frequent in children (5-8 years), than in preadolescents (9-12 years) and adolescents (13-16 years); while separation distress was more common in children and preteens than in teens. According to the American Psychiatric Association (2000), young children do not usually express specific concerns, but as they get older, the concerns tend to become specified; for example, that parents have an accident. Adolescents, on the other hand, may deny separation anxiety, although it is reflected in the limitations to their independent activity, for example, refusing to leave the house.

The main objective of this research was to validate the parents’ version of the Children’s Separation Anxiety Scale (Méndez et al., 2014), which assesses separation anxiety symptoms in pre-adolescence, the life stage with the highest incidence of SAD. To this end, we carried out two studies with independent samples: in Study 1, we performed an exploratory factor analysis of the parent’s version of the scale (CSAS-P); In Study 2, we performed a confirmatory factor analysis, internal consistency, temporal stability, factor invariance and the difference in latent means, as well as the analysis of the differences between the child’s assessment (CSAS) and that of the parents (CSAS- P).

## Study 1

### Materials and Methods

#### Participants

A random cluster sampling was carried out in two provinces in southeastern Spain. The primary units were the comarcas, the secondary units were the schools, and the tertiary units were the classrooms. 1,285 parents, whose children were in 3rd to 6th grade of Primary Education, were recruited from 13 schools. 196 (9.78%) parents were excluded due to errors or omissions in the answers, because they did not give informed consent, or because they were foreigners with significant deficits in the command of Spanish. The sample consisted of 1,089 parents, from 26 to 59 years of age (*M* = 38.57, *SD* = 5.96). Most of the parents were Spanish (89.26%), the rest were non-Spanish European (3.49%), Latin American (3.03%), North African (2.30%), and Asian (1.93%). Regarding the composition, 77.59% of the families were formed by both parents, and 22.41% by a single parent. Concerning educational level, 40.40% had higher education, 32.60% intermediate studies, 24.79% primary studies, and 2.20% did not report this. The socioeconomic status of the families was medium-high or high (28.65%), medium (43.71%), and medium-low or low (27.64%). Children, from 8 to 11 years old (*M* = 9.59, *SD* = 1.11), 51.7% girls, attended public (60.98%), subsidized (30.04%) and private (8.97%) schools. The chi-squared test for homogeneity of the distribution of frequencies indicated that there were no statistically significant differences between the eight groups of age x gender (χ^2^ = 3.12, *df* = 3, *p* = 0.37).

#### Instruments

##### Demographic Form

A short questionnaire was developed to collect data on age, gender, nationality, family structure, educational level, and socioeconomic status.

##### Children’s Separation Anxiety Scale - Parent Version

This is the adaptation for parents of the original scale for children (Méndez et al., 2014), which assesses the frequency of symptoms of separation anxiety in the child. It consists of 20 items and is rated on a five-point Likert-type scale with options 1 (*never or almost never*), 2 (*sometimes*), 3 (*often*), 4 (*very often*), and 5 (*always or almost always*).

#### Procedure

After obtaining permission from the educational authorities, the researchers met with the principals and the heads of the Psychology Department of the selected schools to inform them verbally and in writing of the study objectives, request their authorization, and obtain their collaboration. An informational meeting was held with the parents in which their written consent was requested and the demographic form and the CSAS-P were provided to them, which they had to complete within one week.

#### Ethical Considerations

This study was approved by the Research Ethics Committee of the University of Alicante (Spain), reference number UA-2019-07-10.

#### Statistical Analysis

The underlying structure of the CSAS-P was determined by iterative principal axis factor analysis with oblique rotation because the factors were correlated. Principal axis analysis was used as it was considered, within the ordinary least squares methods, the recommended classical option when the assumption of normality is not fulfilled (Fabrigar et al., 1999). The distribution of the data was explored and some items yielded values of a non-normal distribution. There were 183 participants who left between 1 and 3 items unanswered. The missing data were assigned using the multiple imputation method (Lang and Little, 2018). To interpret the goodness of fit, saturations equal to or greater than 0.35 were taken as a reference. The factors were not forced to equate their number with the expected factors.

Data analyses were performed with the SPSS statistical package, version 20.0.

### Results

**FIGURE 1:**
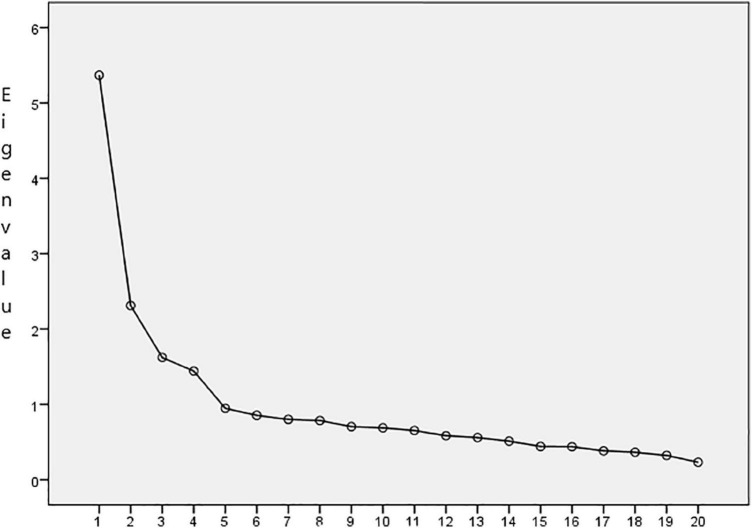
The scree plot.

The Kaiser-Meyer-Olkin measure of sampling adequacy (KMO = 0.86) and Bartlett’s sphericity test (χ^2^ = 6990.87, *df* = 190, *p* < 0.001) showed adequate values. The same four factors were obtained as in the original version for the child, with an eigenvalue greater than one (Kaiser criterion) and with an explained variance of 42.93% (see Figure 1). The factor loadings varied between 0.35 and 0.77 (*M* = 0.59). Factor 1, Worry (Items 10, 13, 16, 18, and 20), 14.80% of the explained variance, is the cognitive component of anxiety that assesses the child’s concern about something bad happening to their parents and/or to them. Factor 2, Opposition (Items 1, 2, 4, 5, and 9), 10.74% of the explained variance, is the behavioral component of anxiety and refers to the child’s actions to avoid or end the situation of being separated from parents. Factor 3, Calm (Items 3, 8, 12, 15, and 19), 9.50% of the explained variance, is a positive factor that expresses the child’s confidence when separated from their parents or away from home. Factor 4, Distress (Items 6, 7, 11, 12, and 14), 7.89% of the explained variance, is the psychophysiological component of anxiety and includes the discomfort experienced by the child when they are separated from their parents (see Table 1).

**TABLE 1:** Exploratory factor analysis.

Items Your son/daughter. [¿Su hijo/a…]	Factor 1	Factor 2	Factor 3	Factor 4
13. Is worried that you or your partner may have an accident? [Está preocupado/a por si usted, o su pareja, sufre un accidente?]	**0.772**	0.176	−0.058	0.017
20. Is worried that he/she might have an accident? [Está preocupado/a por si él/ella sufre un accidente?]	**0.753**	0.116	−0.103	0.155
16. Is worried about something bad happening to him/her? [Está preocupado/a por si a él/ella le sucede algo malo?	**0.752**	0.082	−0.099	0.155
10. Is worried about something bad happening to you or your partner? [Está preocupado/a por si a usted, o a su pareja, le sucede algo malo?]	**0.734**	0.193	−0.081	0.018
18. Is worried about his/her health? [Está preocupado/a por su salud (de él/ella)?	**0.678**	0.051	−0.118	0.128
9. Protests if you or your partner plan to go out at night? [Protesta si usted, o su pareja, planea salir por la noche?]	0.135	**0.733**	−0.118	0.138
2. Protests if you or your partner tell him/her that you are going out? [Protesta si usted, o su pareja, le dice que va a salir?]	0.120	**0.702**	−0.150	0.078
4. Tries to convince you or your partner not to go on a trip? [Intenta convencerle a usted, o a su pareja, de que no se vaya de viaje?]	0.106	**0.672**	−0.158	0.234
5. Cries and protests when he/she is separated from you or your partner? [Llora y protesta cuando está separado de usted o de su pareja?]	0.276	**0.410**	−0.213	0.256
1. Tries to phone you or your partner when you are not with him/her? [Intenta telefonearle a usted, o a su pareja, cuando no está con él/ella?]	0.190	**0.382**	−0.206	0.146
8. Is calm even though he/she can’t phone you or your partner? [Está tranquilo/a aunque no pueda telefonearle a usted o a su pareja?]	−0.141	−0.252	**0.621**	−0.087
3. Is calm even though you or your partner are not with him/her? [Está tranquilo/a aunque usted, o su pareja, no esté con él/ella?]	−0.094	−0.207	**0.593**	−0.105
15. Is calm if he/she goes on a trip without you or your partner? [Está tranquilo/a si se va de viaje sin usted o sin su pareja?]	−0.015	−0.072	**0.560**	−0.146
19. Is he/she calm when is away from home? [Está tranquilo/a si él/ella está lejos de casa?]	−0.156	−0.054	**0.546**	−0.110
12. Is he/she calm when it gets dark and you or your partner are not there? [Está tranquilo/a si se hace de noche y no está usted o su pareja?]	−0.016	−0.084	**0.488**	−0.046
14. Cries when you or your partner say goodbye to him/her at school? [Llora cuando usted, o su pareja, se despide de él/ella en el colegio?]	0.058	0.077	−0.013	**0.703**
11. Feels bad when you or your partner drop him/her off at school? [Se siente mal cuando usted, o su pareja, le deja en el colegio?]	0.054	0.096	−0.082	**0.572**
6. Feels bad at school because you or your partner are not with him/her? [Se siente mal en el colegio porque usted, o su pareja, no está con él/ella?]	0.086	0.165	−0.163	**0.509**
7. Complains of a tummy ache when he/she is separated from you or your partner? [se queja de dolor de barriga cuando se separa de usted o de su pareja?]	0.051	0.299	−0.161	**0.354**
17. Is he/she afraid to eat at school in case he/she might vomit or choke? [Tiene miedo de comer en el colegio por si siente ganas de vomitar o por si se atraganta?]	0.081	0.063	−0.081	**0.352**

*The item loadings of the four factors are in bold to facilitate the reading in Table 1.*

## Study 2

### Materials and Methods

#### Participants

Similar to Study 1, a random cluster sampling was carried out in three provinces in southeastern Spain. 4,271 parent-child dyads were recruited from 43 schools that had not participated in Study 1.470 (11%) parent-child dyads were excluded due to errors or omissions in the answers, because they did not give informed consent, or because they were foreigners with significant deficits in the command of Spanish. The sample consisted of 3,801 parent-child dyads. The age range of the parents was 25-57 years (*M* = 37.23, *SD* = 5.48). The nationality of the families was Spanish (87.13%), non-Spanish European (4.13%), Latin American (3.05%), or other (5.68%). The children lived with both parents (74.40%), with the mother alone (20.68%), or with the father alone (4.92%). Regarding the parents’ educational level, 39.17% had higher education, 33.78% intermediate education, and 27.05% primary education or a lower level. The socioeconomic status of most of the families was medium (47.83%), and the rest were medium-low or low (26.89%) and medium-high or high (25.28%).

Test-retest reliability was calculated with 590 parents randomly selected from the sample, who completed the CSAS-P again four weeks later.

Children, from 8 to 11 years old (*M* = 9.50, *SD* = 1.10), 50.2% girls, attended public (60.98%), subsidized (30.04%) and private (8.97%) schools. The Chi-square test of homogeneity of the frequency distribution revealed that there were no statistically significant differences between the eight age x gender groups (χ^2^ = 2.34, *df* = 3, *p* = 0.50).

#### Instruments

The demographic form and the CSAS-P were completed by the parents: 72.11% by the mother, 16.23% by the father, and 11.65% by both.

The children answered the CSAS (Méndez et al., 2014). The coefficients omega were adequate in this study: CSAS (0.89), Worry (0.78), Opposition (0.72), Calm (0.73), and Distress (0.70). The correlation with other measures of separation anxiety is high: *r* = 0.71 with the Separation Anxiety Assessment Scale (SAAS; Eisen and Schaefer, 2005), *r* = 0.62 with the Separation Anxiety subscale of the Screen for Child Anxiety-Related Emotional Disorders (SCARED; Birmaher et al., 1997; Spanish version, Vigil-Colet et al., 2009), and *r* = 0.61 with Separation Anxiety Disorder subscale from the Spence Children’s Anxiety Scale (SCAS; Spence, 1997; version Spanish, Orgilés et al., 2012a).

#### Procedure

The process with the parents was similar to that of Study 1. After obtaining parental consent, the children collectively completed the CSAS in the classroom during school hours.

#### Statistical Analysis

First, the internal structure of the CSAS-P was contrasted using four confirmatory factor analyses (CFAs): null model (0 factors), 1-factor model, 4-uncorrelated factor model, and 4-correlated factor model from Study 1. As the Mardia multivariate kurtosis coefficient was very high (405.23), exceeding the value 5 and revealing that the data did not fit the multivariate normal distribution (Bentler, 2006), the robust maximum likelihood method was used. As the use of several indices is recommended to evaluate the fit of a structural model (Weston and Gore, 2006; Kline, 2013), in addition to the Satorra-Bentler scaled chi-square statistic (S-Bχ^2^), the following indices were used: Robust Root Mean Square Error of Approximation (R-RMSEA): < 0.05 excellent fit, < 0.08 acceptable fit; Standardized Root Mean-squared Residual (SRMR): < 0.05 good fit, close to 0.08 acceptable fit; Robust Comparative Fit Index (R-CFI): ≥ 0.95 good fit, ≥ 0.90 acceptable fit; and Tucker-Lewis Index (TLI): ≥ 0.90 acceptable fit (Hu and Bentler, 1999; Brown, 2006). The reliability of the CSAS-P was calculated using Cronbach’s alpha coefficient of internal consistency and Pearson’s product-moment correlation coefficient of temporal stability.

Second, measurement invariance and structural invariance were examined as a function of the age and gender of the child and the parent who had completed the CSAS-P, using multigroup confirmatory factor analysis (MGCFA) to confirm the invariance of the model that would have obtained better fit indices in the previous step. Again, the Mardia coefficients were high: 170.67 (8 years), 180.36 (9 years), 196.84 (10 years), and 240.27 (11 years); 314.78 (boys), and 255.51 (girls); 333.66 (mothers), 171.48 (fathers) and 124.60 (both), so robust maximum likelihood estimators were used to fit the measurement model (Satorra and Bentler, 2001), proceeding according to a series of hierarchical steps (Byrne, 2006, 2008; Liu et al., 2015; Samuel et al., 2015). In Model 0, no restrictions were set on configural invariance; in Model 1, factor load restrictions were imposed for metric invariance; in Model 2, restrictions were imposed of the factor loadings and the intercepts of the variables for scalar invariance or strong invariance; in Model 3, restrictions were imposed of the factor loadings, the intercepts of the variables, and the variances and co-variances of the errors for the strict invariance; in Model 4, the variances and co-variances of the factors in Model 2 were matched to assess structural invariance. The fit of the models was assessed using the above-mentioned indices (R-RMSEA, SRMR, R-CFI, and TLI) and the equivalence of the models through the change in the Satorra-Bentler scaled chi-square statistic (ΔS-Bχ^2^) with *p* > 0.05 and in the Comparative Fit Index (ΔCFI) with differences > −0.01 (Cheung and Rensvold, 2002).

Third, the critical ratio (CR) was used to assess the existence of significant differences in the latency of means in parents across age, gender of the children and the parent who completed the scale (significant difference, -1.96 > CR > 1.96; Tsaousis and Kazi, 2013). The effect size was calculated using Cohen’s *d* statistic (Fritz et al., 2012). Regarding the age of the child, the scores of the parents with younger children in each of the three comparisons that were made were set to zero (8, 9, and 10 years, respectively). Regarding gender, the scores of the parents of the boys were set to zero to compare them with the scores of the parents of the girls. Regarding the person who completed the questionnaires, two comparisons were made in which the mothers and fathers, respectively, were taken as reference.

Finally, Student’s *t-*test was used to analyze the differences between the scores of the parents and the children on the scale, and the Pearson correlation (interclass correlation coefficient) was used to compare the scores of the child and the parents.

The analyses described were carried out with the SPSS program, version 20, and with the EQS program, version 6.1.

## Results

### Confirmatory Factor Analysis

**FIGURE 2:**
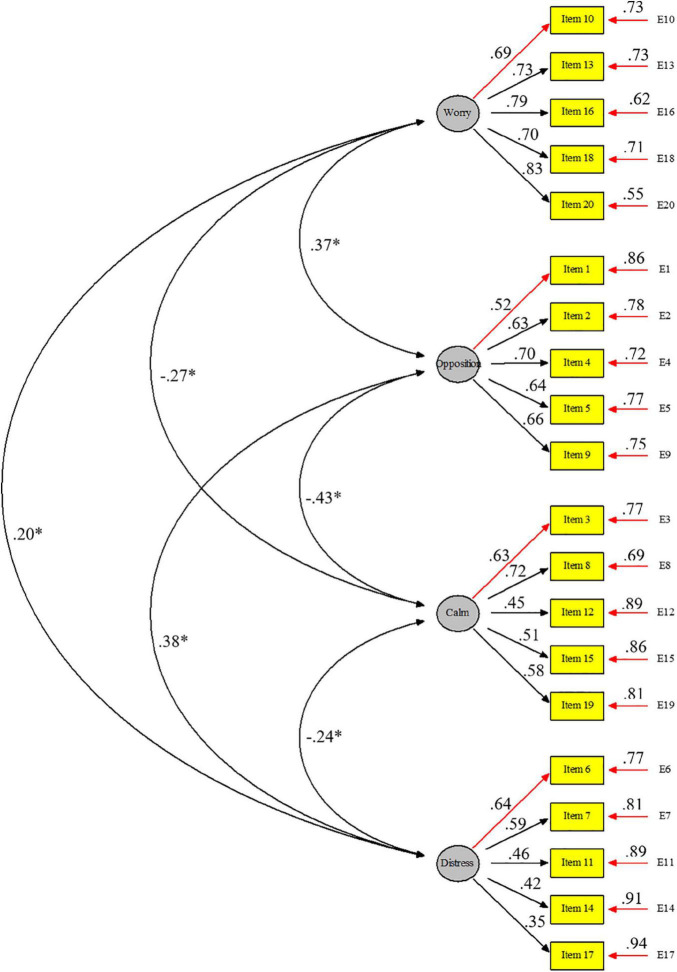
Graphic representation of the 4-factor model of the CSAS-P.

The model with four correlated factors was the one that obtained the best fit and adequate indices (Table 2). Table 3 shows the correlation coefficients between the factors and with the total score of the CSAS-P. Graphic representation of the 4-factor model of the CSAS-P with the factor loadings, the associated standard errors and the correlations among factors are shown in Figure 2.

**TABLE 2:** Goodness-of-fit indexes of the statistic models of the CSAS-P.

	S-Bχ^2^	*df*	R-RMSEA 90% CI	SRMR	R-CFI	TLI
Null model	13,341.35	190	0.135 [0.133, 0.137]	0.243	0.000	0.000
1-factor model	6,447.30	170	0.099 [0.097, 0.101]	0.114	0.523	0.467
4-factor model (uncorrelated)	2,384.95	190	0.060 [0.057, 0.062]	0.154	0.831	0.806
4-factor model (correlated)	876.47	159	0.034 [0.032, 0.037]	0.039	0.945	0.935

*S-Bχ^2^ = Satorra-Bentler scaled χ^2^; df = degrees of freedom; R-RMSEA = robust root mean square error of approximation; CI = confidence interval; SRMR = standardized root mean square residual; R-CFI = robust comparative fit index; TLI = Tucker Lewis Index.*

**TABLE 3:** Correlation matrix among factors and with CSAS-P total score.

	1. Worry	2. Opposition	3. Calm	4. Distress	Total
(1). Worry	————	————	————	————	————
(2). Opposition	0.37*	————	————	————	————
(3). Calm	−0.27*	−0.43*	————	————	————
(4). Distress	0.20*	0.38*	−0.24*	————	————
Total	0.77*	0.75*	−0.72*	0.48*	————

*p ≤ 0.001.

### Reliability

The coefficients omega were: 0.92 for the CSAS-P, 0.86 for Factor 1 Worry, 0.77 for Factor 2 Opposition, 0.72 for Factor 3 Calm, and 0.62 for Factor 4 Distress. The test-retest reliability coefficients were: 0.72 for the CSAS-P, 0.68 for Factor 1 Worry, 0.68 for Factor 2 Opposition, 0.58 for Factor 3 Calm, and 0.56 for Factor 4 Distress.

### Factor Invariance Across Child’s Age and Gender

Tables 4–6 show that the invariance models analyzed presented a good fit according to the indices used. The requirements that no ΔS-Bχ^2^ value was statistically significant and that ΔCFI values were greater than −0.01 were also met. Therefore, the measurement and structure invariance were confirmed for the CSAS-P of 4 correlated factors based on the age and gender of the child and the parent who completed the scale.

**TABLE 4:** Goodness-of-fit indexes for CSAS-P depending on child’s age.

	χ^2^	S-Bχ^2^	*df*	R-RMSEA	SRMR	R-CFI	TLI	Δ S-Bχ^2^ (Δ *df, p)*	Δ R-CFI
8 year old	471.966	327.3785	159	0.036 [0.030, 0.041]	0.049	0.946	0.935		
9 year old	457.773	337.0742	159	0.035 [0.029, 0.040]	0.045	0.944	0.933		
10 year old	482.639	329.4643	159	0.033 [0.028, 0.038]	0.042	0.950	0.940		
11 year old	547.236	373.0619	159	0.036 [0.031, 0.041]	0.047	0.938	0.925		
Model 0	1,959.613	1,367.6108	636	0.017 [0.016, 0.019]	0.046	0.944	0.934		
Model 1	2,089.829	1,417.7815	684	0.017 [0.016, 0.018]	0.049	0.944	0.938	64.51 (48, 0.066)	0.000
Model 2	2,159.964	1,500.0567	744	0.017 [0.015, 0.018]	0.049	0.943	0.933	66.72 (60, 0.257)	−0.001
Model 3	2,602.250	1,490.2203	819	0.015 [0.014, 0.016]	0.059	0.943	0.935	92.44 (75, 0.084)	0.000
Model 4	2,264.225	1,531.2541	774	0.016 [0.015, 0.017]	0.059	0.943	0.936	42.71 (30, 0.062)	0.000

*Model 0 = free model; Model 1 = Model 0 with factor loadings; Model 2 = Model 1 with intercepts; Model 3 = Model 2 with error variances and co-variances; Model 4 = Model 2 with factor variances and co-variances; S-Bχ^2^ = Satorra-Bentler scaled χ^2^; df = degrees of freedom; R-RMSEA = robust root mean square error of approximation; CI = confidence interval; SRMR = standardized root mean square residual; R-CFI = robust comparative fit index; TLI = Tucker Lewis Index.*

**TABLE 5:** Goodness-of-fit indexes for CSAS-P depending on child’s gender.

	χ^2^	S-Bχ^2^	*df*	R-RMSEA	SRMR	R-CFI	TLI	Δ S-Bχ^2^ (Δ *df, p)*	Δ R-CFI
Boys	725.527	496.9740	159	0.034 [0.030, 0.037]	0.044	0.942	0.931		
Girls	810.379	569.2967	159	0.037 [0.034, 0.040]	0.040	0.943	0.931		
Model 0	1,535.906	1,065.3573	318	0.025 [0.023, 0.027]	0.042	0.942	0.931		
Model 1	1,559.681	1,066.0674	334	0.024 [0.022, 0.026]	0.043	0.943	0.935	12.60 (16, 0.701)	0.001
Model 2	1,589.689	1,096.3763	354	0.024 [0.022, 0.026]	0.043	0.943	0.934	24.37 (20, 0.226)	0.000
Model 3	1,739.847	1,062.0521	379	0.023 [0.021, 0.024]	0.045	0.944	0.936	34.88 (25, 0.090)	0.001
Model 4	1,625.528	1,102.8524	364	0.024 [0.022, 0.025]	0.049	0.943	0.936	15.43 (10, 0.117)	0.000

*Model 0 = free model; Model 1 = Model 0 with factor loadings; Model 2 = Model 1 with intercepts; Model 3 = Model 2 with error variances and co-variances; Model 4 = Model 2 with factor variances and co-variances; S-Bχ^2^ = Satorra-Bentler scaled χ^2^; df = degrees of freedom; R-RMSEA = robust root mean square error of approximation; CI = confidence interval; SRMR = standardized root mean square residual; R-CFI = robust comparative fit index; TLI = Tucker Lewis Index.*

**TABLE 6:** Goodness-of-fit indexes for CSAS-P depending on the parent who fulfilled the scale.

	χ^2^	S-Bχ^2^	*df*	R-RMSEA	SRMR	R-CFI	TLI	Δ S-Bχ^2^ (Δ *df, p)*	Δ R-CFI
Mother	885.429	614.6135	159	0.032 [0.030, 0.035]	0.037	0.952	0.943		
Father	412.857	273.6021	159	0.034 [0.027, 0.040]	0.053	0.941	0.929		
Parent	366.342	277.8189	159	0.041 [0.032, 0.048]	0.061	0.926	0.912		
Model 0	1,664.650	1,170.2133	477	0.020 [0.018, 0.021]	0.051	0.948	0.938		
Model 1	1,738.245	1,178.2872	509	0.019 [0.017, 0.020]	0.055	0.950	0.944	32.55 (32, 0.440)	0.002
Model 2	1,787.720	1,238.8076	549	0.019 [0.017, 0.020]	0.055	0.950	0.941	47.82 (40, 0.185)	0.000
Model 3	2,026.278	1,218.9934	599	0.017 [0.015, 0.018]	0.061	0.955	0.948	58.63 (50, 0.189)	0.005
Model 4	1,813.650	1,252.6510	569	0.018 [0.017, 0.020]	0.060	0.950	0.941	16.45 (20, 0.689)	0.000

*Model 0 = free model; Model 1 = Model 0 with factor loadings; Model 2 = Model 1 with intercepts; Model 3 = Model 2 with error variances and co-variances; Model 4 = Model 2 with factor variances and co-variances; S-Bχ^2^ = Satorra-Bentler scaled χ^2^; df = degrees of freedom; R-RMSEA = robust root mean square error of approximation; CI = confidence interval; SRMR = standardized root mean square residual; R-CFI = robust comparative fit index; TLI = Tucker Lewis Index.*

### Latent Mean Differences Across Child’s and Parent’s Age and Gender

Regarding the age of the child, the statistics of the latent mean structures were adequate. Taking 8 years as a reference: S-B χ^2^ = 1555.156, *df* = 732, *p* < 0.000; R-RMSEA = 0.017, CI = 0.016, 0.018; SRMR = 0.049; R-CFI = 0.944; TLI = 0.933; 9 years as a reference: S-B χ^2^ = 1171.501, *df* = 541, *p* < 0.000; R-RMSEA = 0.020, CI = 0.018, 0.021; SRMR = 0.049; R-CFI = 0.942; TLI = 0.931; 10 years as a reference: S-Bχ^2^ = 754.572, *df* = 350, *p* < 0.000; R-RMSEA = 0.024, CI = 0.022, 0.026; SRMR = 0.048; R-CFI = 0.942, TLI = 0.931. In relation to the child’s gender, adequate fit indices were also obtained, taking as reference the boys: S-Bχ^2^ = 1140.901, *df* = 350, *p* < 0.000; R-RMSEA = 0.024, CI = 0.023, 0.026; SRMR = 0.045; R-CFI = 0.943; TLI = 0.932. Finally, the fit indices referring to the parent who had answered the CSAS-P were adequate. Taking the mothers as a reference: S-Bχ^2^ = 1253.279, *df* = 541, *p* < 0.000; R-RMSEA = 0.019, CI = 0.017, 0.020; SRMR = 0.056; R-CFI = 0.950; TLI = 0.940; taking the fathers as reference: S-Bχ^2^ = 598,171, *df* = 350, *p* < 0.000; R-RMSEA = 0.026, CI = 0.022, 0.029; SRMR = 0.060; R-CFI = 0.937; TLI = 0.924.

As can be seen in Table 7, the age differences were scarce and of minimal size (*d* < 0.28); the general pattern was that older children scored higher on Worry and younger children on Distress from. Girls scored higher on all factors, except for Calm, although the gender difference was small. The differences depending on the parent who completed the CSAS-P were also small and there was no clear pattern.

**TABLE 7:** Scores of latent mean differences across child’s age and gender, and parent.

	1.Worry	2.Opposition	3.Calm	4.Distress
***8 year old (reference)***				
9 year old Mean estimate Standard error Critical ratio	−0.040 0.052 −0.772	−0.055 0.036 −1.524	−0.008 0.043 −0.177	0.006 0.020 0.296
10 year old Mean estimate Standard error Critical ratio	0.073 0.053 1.384	0.027 0.038 0.701	−0.030 0.043 −0.709	0.012 0.021 0.586
11 year old Mean estimate Standard error Critical ratio	0.172 0.053 3.242* (*d* = 0.150)	−0.035 0.036 −0.970	−0.026 0.042 −0.624	−0.036 0.019 −1.919
***9 year old (reference)***				
10 year old Mean estimate Standard error Critical ratio	0.112 0.051 2.213* (*d* = 0.101)	0.085 0.036 2.346* (*d* = 0.107)	−0.023 0.041 −0.570	0.007 0.020 0.337
11 year old Mean estimate Standard error Critical ratio	0.211 0.051 4.130* (*d* = 0.187)	0.022 0.034 0.637	−0.019 0.041 −0.472	−0.040 0.018 −2.290* (*d* = 0.103)
***10 year old (reference)***				
11 year old Mean estimate Standard error Critical ratio	0.096 0.050 1.924	−0.063 0.037 −1.701	0.004 0.040 0.087	−0.042 0.019 −2.220* (*d* = 0.099)
***Boys (reference)***				
Girls Mean estimate Standard error Critical ratio	0.156 0.037 4.230* (*d* = 0.138)	0.207 0.026 7.986* (*d* = 0.261)	−0.197 0.030 −6.585* (*d* = 0.215)	0.042 0.013 3.093* (*d* = 0.100)
***Mothers (reference)***				
Fathers Mean estimate Standard error Critical ratio	0.032 0.051 0.636	−0.102 0.030 −3.399* (*d* = 0.118)	0.039 0.039 0.989	−0.029 0.017 −1.718
Parents Mean estimate Standard error Critical ratio	0.367 0.063 5.864* (*d* = 0.209)	−0.029 0.040 −0.729	0.051 0.045 1.130	−0.056 0.017 −3.204* (*d* = 0.114)
***Fathers (reference)***				
Parents Mean estimate Standard error Critical ratio	0.339 0.076 4.453* (*d* = 0.273)	0.070 0.046 1.512	0.011 0.055 0.197	−0.024 0.020 −1.194

*p ≤ 0.05.

### Parent-Child Agreement

The correlation coefficient between CSAS (child) and CSAS-P (parents) was *r* = 0.28 and between the respective factors on both scales: *r* = 0.15 Worry, *r* = 0.31 Opposition, *r* = 0.13 Calm, and *r* = 0.17 Distress. These coefficients, although significant, were low (0.10 ≤ *r* ≤ 0.30), except for the Opposition factor, which was medium (0.30 ≤ *r* ≤ 0.50) (Cohen, 1988). Parents scored significantly lower than the child on all factors on the scale, except for Calm. Disagreement was large (*d* ≥ 0.80) in Worry and Calm, moderate (0.50 ≤ *d* < 0.80) in Distress, and small (0.20 ≤ *d* < 0.50) in Opposition. The difference was greater than a point and a half in three items related to the child’s concern for their health and well-being: Item 18 (“Is your child worried about his/her health?” [1.96]), Item 16 (“Is your child worried about something bad happening to them?” [1.73]), and Item 20 (“Is your child worried about having an accident?” [1.51]). On the contrary, the highest degree of agreement between the child and the parents was found in Item 2 (“Does your child protest if you or your partner, tell him that you are going out?” [−0.06]), Item 7 (“Does your child complain of a tummy ache when he/she is separated from you or her partner? [0.23]), and Item 14 (“Does your child cry when you or your partner say goodbye to him at school? [0.23]) (see Table 8).

**TABLE 8:** Mean and standard deviation of child on CSAS and parent on CSAS-P.

	Child		Parent		Statistical Significance		
*Factors*	*M*	*SD*	*M*	*SD*	*t_3_,_080_*	*p*	*d*
1. Worry	20.91	4.79	12.87	6.24	67.84	< 0.001	1.44
2. Opposition	11.14	4.75	9.00	4.13	25.19	< 0.001	0.48
3. Calm	10.62	5.06	15.10	5.08	41.11	< 0.001	0.88
4. Distress	7.31	3.60	5.69	1.85	26.50	< 0.001	0.56
Total Score	54.46	12.01	38.20	12.43	68.34	< 0.001	1.33

*M: Mean; SD: Standard deviation.*

## Discussion

**FIGURE 3:**
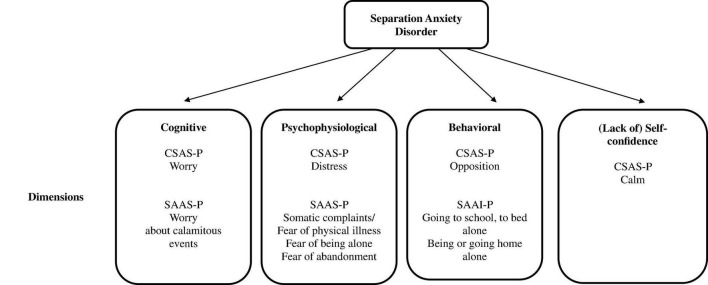
Similarities and differences among SAD scales for parents.

The CSAS-P is based on the three-dimensional theory of Lang (1968), which states that anxiety manifests through three related systems, giving rise to different response profiles depending on the predominant system: cognitive if concern has greater weight, psychophysiological if discomfort predominates, and behavioral if the most relevant aspect is escape/avoidance. Martínez-Monteagudo et al. (2012) state that it is appropriate to evaluate these three dimensions to plan the treatment. In this sense, the CSAS-P represents a contribution to the existing instruments, because the SAAI-P is limited to escape/avoidance behavior, a dimension that is not explicitly addressed in the SAAS-P (Figure 3). On another hand, the psychologists who assessed the original bank of items recommended including positive items to control the tendency to respond negatively and, contrary to expectations, when inverting the score, the positive items were not distributed among the negative factors, but rather emerged in a new factor, Calm (Méndez et al., 2008). In studies on the Personal Report of Confidence as Speaker (Paul, 1966) in the adolescent population, our research team found that confidence did not equate to a low level or absence of fear, but instead, self-confidence referred to the enjoyment of speaking. In other words, the experience was not only not scary or neutral, but reinforcing (Méndez et al., 1999, 2004).

Internal consistency for the CSAS-P and the Worry factor was good (Cronbach’s **α >** 0.80) and adequate for the other factors (Cronbach’s **α >** 0.70), values similar to those obtained with the SAAI-P, (0.75 **≤ α ≤** 0.87) and SAAS-P (0.70 **≤ α ≤** 0.84). The temporal stability of the CSAS-P was adequate, although it presented some deficiencies in the Calm and Distress factors (0.55** ≤** *r*** ≤** 0.65). It should be investigated whether the joint completion of the scale by both parents introduces a source of variability in the answer in a significant proportion of cases because the degree of agreement between the mother and the father on the child’s internalized problems is modest (Stanger and Lewis, 1993).

Unlike SAAI-P and SAAS-P, the CSAS-P focuses on pre-adolescence, “a neglected population” (Cartwright-Hatton et al., 2006). A study with schoolchildren revealed that, from the age of 11, there was a generalized decrease in excessive fear, defined as twice the standard deviation above the mean in the Inventory of Fears, by Sosa et al. (1993), both on the intensity and the number of excessive fears; the fear of being separated from the parents increased notably in pre-adolescence: 4.48% (7 years), 9.76-11.11% (8-11 years), 5.10% (12 years) (Méndez et al., 2003).

Not only the increase in separation anxiety in pre-adolescence justifies the development of an instrument for this age group, but also the evolution of its manifestations. González (2003) analyzed the parents’ responses to the Early Onset Separation Anxiety Questionnaire, finding three dimensions: separation anxiety due to the loss or harm of a loved one (e.g., “If you or your partner have been admitted to the hospital, has your child shown excessive signs of anxiety?”); sleep-related separation anxiety (e.g., “If your child wakes up during the night, does he/she call you insistently and you have to go to his/her room to calm him/her down?”); and separation anxiety about everyday events (e.g., “If you are separated from your child to attend a social event [dinner, wedding, etc.], is your child eager for you to come home or does he/she feel the urge to phone you?”). That is, the dimensions referred to the situation (variable E), not to the reaction (variable R).

Our findings on age and gender differences in separation anxiety are consistent with the literature on the subject. The symptoms of separation anxiety diminish with age. Compton et al. (2000) found significantly higher levels in a community sample in the 8-12-year-old group than in the 13-19-year-old group. However, when the age range is reduced, the differences are usually small. The only difference of medium size that Orgilés et al. (2011) found was between the extreme ages of the recruited school sample, 8 and 11 years; younger children scored higher, especially in discomfort. Similarly, in the general population, separation anxiety symptoms are more frequent in the female gender (Orgilés et al., 2003), although again, this difference is small (Orgilés et al., 2012b).

The correlation between the child’s and parents’ scores was low. Studies of other separation anxiety scales with community pre-adolescent samples show similar results: *r* = 0.26 SAAS (Orenes, 2015), *r* = 0.36 MASC (Baldwin and Dadds, 2007), *r* = 0.27 SCARED (Cosi et al., 2010), and *r* = 0.16 SCAS (Ishikawa et al., 2014), data that corroborate the conclusion that “in general, parent-child and parent-parent concordance is low for internalizing symptoms, especially for domains that are relatively less observable by parents” (March and Parker, 2004, p. 48). Parent-child disagreement was greater in concern and lower in behaviors such as protesting, complaining, or crying, consistent with the greater degree of agreement in observable symptoms than in unobservable ones (Comer and Kendall, 2004).

Our research was carried out with a school population. Future studies should examine the psychometric properties of the CSAS-P with clinical samples, where parent-child agreement is usually higher. Arendt et al. (2014) obtained correlation coefficients of 0.45 and 0.60 for the SCAS Separation Anxiety Scale both in community and clinical samples.

In our study, carried out within the framework of the multi-source evaluation, and the child and the parents (or the main attachment figures) both participated but the evaluation was reduced to a single instrument. Future research should overcome this limitation and calculate the convergent validity of the CSAS-P with other measures of separation anxiety, as well as the sensitivity and specificity of the scale through a clinical interview.

## Data Availability Statement

The datasets presented in this article are not readily available because Institutional regulations. Requests to access the datasets should be directed to JG-F, josemagf@ua.es.

## Ethics Statement

The studies involving human participants were reviewed and approved by University of Alicante. The patients/participants provided their written informed consent to participate in this study.

## Author Contributions

All authors participated in the design of the study, the analysis and interpretation of the data, the writing of the initial version of the manuscript, and approval of the final version.

## Conflict of Interest

The authors declare that the research was conducted in the absence of any commercial or financial relationships that could be construed as a potential conflict of interest.

## Publisher’s Note

All claims expressed in this article are solely those of the authors and do not necessarily represent those of their affiliated organizations, or those of the publisher, the editors and the reviewers. Any product that may be evaluated in this article, or claim that may be made by its manufacturer, is not guaranteed or endorsed by the publisher.
